# Intraoperative Treatment of Duct of Luschka during Laparoscopic Cholecystectomy: A Case Report and Revision of Literature

**DOI:** 10.1155/2018/9813489

**Published:** 2018-12-17

**Authors:** Luigi Masoni, Leandro Landi, Riccardo Maglio

**Affiliations:** ^1^Universitary Researcher Department General Surgery Sapienza University of Rome, Italy; ^2^University of Naples Vanvitelli, Italy; ^3^Department of General Surgery, Hospital Veris Delli Ponti, Scorrano, Italy

## Abstract

**Background:**

Bile leakage still remains a serious complication during cholecystectomies. In limited cases, this complication may occur from injury of the so-called ducts of Luschka. These rare ducts are usually discovered intraoperatively, and their presence poses the risk of bile injury and clinically significant bile leak.

**Presentation Case:**

We present a unique case of a 59-year-old male patient with acute cholecystitis. After removal of the gallbladder, thorough inspection of the hepatic bed was made and a little bile leak was identified from a duct of Luschka 1 cm away from the gallbladder hilum. We report on the use of endoscopic QuickClip Pro® clips (Olympus Medical Systems Corp., Tokyo, Japan) to avoid further more invasive treatment.

**Discussion:**

Endoscopic retrograde cholangiopancreatography with sphincterotomy played a crucial role for diagnosis and treatment of bile leaks with success rate near 94%. Many authors have argued the role of relaparoscopy, Diagnosis may be intraoperatively but this option does not seem to occur very often; in fact, there is a lack of data in literature.

**Conclusion:**

This is the first case report of bile leak from duct of Luschka treated during the cholecystectomies with endoscopic clip.

## 1. Introduction

Bile leakage represent an important and sometimes life-threatening postoperative complication of open as well as laparoscopic cholecystectomy [1]. The incidence varies between 0.2 and 2%. In limited cases this complication may occur from injury of the so-called ducts of Luschka; this event can be identified either intraoperatively or postoperatively [2]: intraoperative recognition is rare because of the small calibre of the ducts of Luschka but requires immediate resolution to avoid further more invasive treatment. We report on the use of endoscopic QuickClip Pro® clips (Olympus Medical Systems Corp., Tokyo, Japan) in a case where the quality of liver tissue did not allow safe suturing of the duct. This case report is in line with the SCARE guidelines [3].

## 2. Case Report

**Figure 1:**
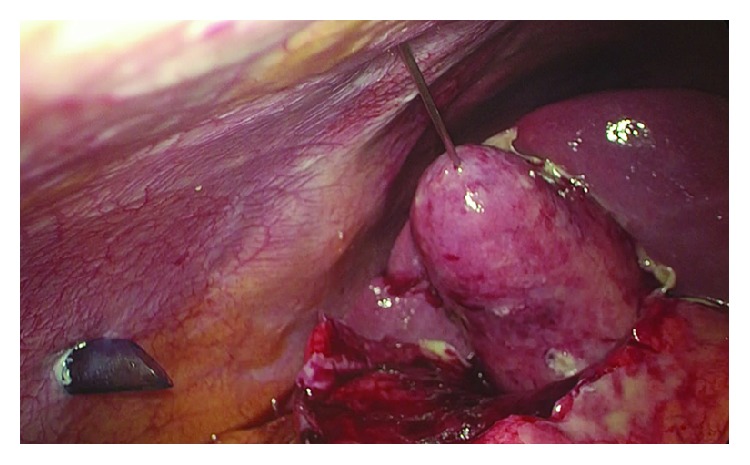
Evacuation of empyematous gallbladder.

**Figure 2:**
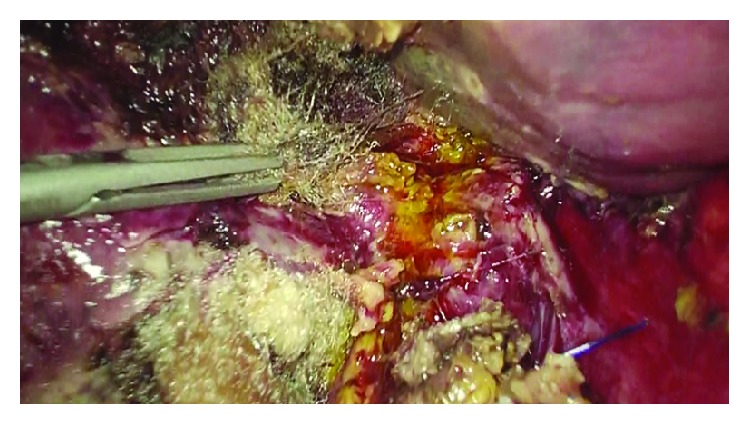
Duct of Luschka.

**Figure 3:**
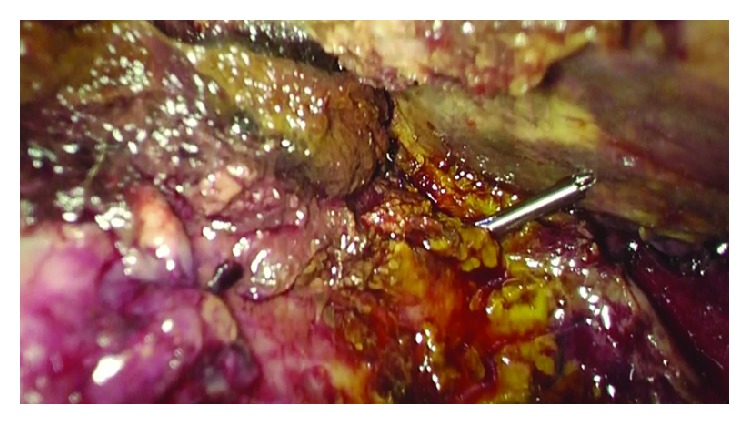
Closing the duct of Luschka with QuickClip Pro® clips.

A 59-year-old male was admitted to our ward with abdominal pain in the right upper quadrant, nausea, and vomiting; the symptoms had started about 24 hours before. Physical examination showed tenderness of the abdomen, positive Murphy's sign, negative Blumberg's sign. On admission, blood test showed WBC count of 17.200/mm^3^, whereas liver function tests, lipase, and amylase levels all resulted as normal. Abdominal ultrasound showed a single gallstone impacted in the infundibulum of a dilated gallbladder, with a thick and inflamed wall. The patient underwent emergency laparoscopic cholecystectomy. A three-trocar technique was used inserting the cannulas in the umbilicus (10 mm), subxiphoid (5 mm), and right lateral subcostal margin (5 mm). Preliminary evacuation of empyematous gallbladder was performed by a percutaneous 21-gauge needle (Figure 1). A fundus first approach was elected because of the severe inflammation of tissues surrounding the gallbladder and its hilum. The procedure lasted 45 minutes with repeated use of bipolar energy to control bleeding from the gallbladder bed. After removal of the gallbladder, thorough inspection of the hepatic bed was made and a little bile leak was identified from a duct of Luschka 1 cm away from the gallbladder hilum (Figure 2). Direct suture with 5/0 PDS was attempted first but failed because of the poor quality of the inflamed hepatic tissue. In order to avoid any deeper suture that would involve major hepatic vessels due to the proximity with the hepatic hilum, an alternative technique was chosen. We inserted a QuickClip Pro® clip (Olympus Medical Systems Corp., Tokyo, Japan) through the subxiphoid trocar. This endoscopic device was directed by means of a Johann clamp inserted through the right subcostal trocar to securely close the duct of Luschka (Figure 3). An abdominal drainage was left in place for 24 hours, and the patient was discharged on the second postoperative day.

## 3. Discussion

As lately described by Schnelldorfer and collegues [4], the so-called “ducts of Luschka” are indeed a variegate group of rare anatomical variations (*subvesical bile duct*) categorized in four types: segmental or sectorial, accessory, hepaticocholecystic, and aberrant bile ducts. They generally drain into the right hepatic duct or the common hepatic duct, but variations are ordinary [4]. They commonly do not drain in the gallbladder [5]. Despite that, some authors showed perplexity due to the lack of universally accepted criteria [6]. In fact, in the literature, they are often called indifferently accessory biliary ducts, subvesicular ducts, or supravesicular ducts [6]. Nevertheless, we can generally assert that the ducts of Luschka are a topographic description of additional bile ducts in contact with the gallbladder fossa [4]. Otherwise, aberrant bile ducts are a network of microscopic bile ducts within the connective tissue of the gallbladder wall [4].

Regarding the etiology, we can assert that two are the main hypothesis: congenital or inflammatory [4, 7]. The first hypothesis prompts to an embriogenetic development during the third-fourth week of gestation [7]. Otherwise, some authors think it would be secondary to a parenchymal remodelling [7] or inflammation due to hypertrophy of hepatic branches [4]. The prevalence of such anatomical variations is certainly very low but not available because of the lack of information in literature [8]. Bile leaks are reported in 0.2-2% of all patients treated with laparoscopic cholecystectomies [9]. Frequency of leakage of the ducts of Luschka represents 4.4% of all iatrogenic biliary duct injuries and 15% of type A injuries (according to the “Strasberg classification system” [10]). However, clinical complications related to bile leaks are about 0.4-1.2% [4, 11]. Diagnosis may be intraoperatively (as shown in our case report), but this option does not seem to occur very often; in fact, there is a lack of data in literature; but it may certainly be postoperative: MRCP has a sensitivity of 66%, DIC-CT shows a sensitivity of 100% instead [10]. Unluckily, these imaging techniques are not a routine procedure [12] because of the increase of overall costs. The use of relaparoscopy seems to be controversial: some authors describe it as mandatory [13], some others assert that this option would lead to some more complications and a worse treatment outcome [14]. Almost all subvesical bile duct leaks could receive an endobiliary stent placement [5] or sphincterotomy by ERCP. Doubtfully, relaparoscopy of the patient can be attempted.

Bile loss from subvesical bile duct injury is commonly diagnosed within the first postoperative week [5]. Patient may complain of specific symptoms such as abdominal pain or signs such as tenderness and fever [9]. Increase of ALP and bilirubin may also occur. Despite that, an underestimation of this nonspecific symptomatology could lead to serious consequences and to more serious complications (biliary peritonitis with subsequent sepsis) [12]. Nevertheless, in the literature, we also found cases in which bile loss and consequent symptomatology is suspected weeks after cholecystectomy [15] and cases where no patient referred any symptoms at all [12]. Generally, subvesical bile duct injuries cause a minor symptomatology comparing to a major undetected bile leak that might provoke peritonitis, biliomas, and septic shock in a shorter period of time [16]. All symptoms mentioned certainly need immediate further investigation. Postoperatively, a subvesical duct injury may be diagnosed via fistulography [12].

## 4. Conclusion

Bile leakage still remains a serious complication during cholecystectomies. Intraoperative treatment does not seem to occur at all in the international. Anyway, stent placement or sphincterotomy seems to be the most used therapeutic procedure. Relaparoscopy is not universally accepted because of the risk of postoperation complications paved by some authors [14]. Nevertheless, a careful procedure (in terms of preoperative imaging and meticulous technique) should be carried out as a topic for every laparoscopic operation in order to avoid even such rare but dangerous complications.

## Additional Points

*Guarantor.* Our guarantor is Prof. Luigi Masoni. He conceived the idea of this case report and wisely revised it.
